# The Role of Bmp- and Fgf Signaling Modulating Mouse Proepicardium Cell Fate

**DOI:** 10.3389/fcell.2021.757781

**Published:** 2022-01-04

**Authors:** Carlos Garcia-Padilla, Francisco Hernandez-Torres, Estefania Lozano-Velasco, Angel Dueñas, Maria del Mar Muñoz-Gallardo, Isabel S. Garcia-Valencia, Lledó Palencia-Vincent, Amelia Aranega, Diego Franco

**Affiliations:** ^1^ Cardiovascular Development Group, Department of Experimental Biology, University of Jaen, Jaen, Spain; ^2^ Department of Anatomy, Embryology and Zoology, School of Medicine, University of Extremadura, Badajoz, Spain; ^3^ Fundación Medina, Granada, Spain; ^4^ Department of Biochemistry and Molecular Biology, School of Medicine, University of Granada, Granada, Spain

**Keywords:** Bmp, Fgf, proepicardium, cell fate, heart development

## Abstract

Bmp and Fgf signaling are widely involved in multiple aspects of embryonic development. More recently non coding RNAs, such as microRNAs have also been reported to play essential roles during embryonic development. We have previously demonstrated that microRNAs, i.e., miR-130, play an essential role modulating Bmp and Fgf signaling during early stages of cardiomyogenesis. More recently, we have also demonstrated that microRNAs are capable of modulating cell fate decision during proepicardial/septum transversum (PE/ST) development, since over-expression of miR-23 blocked while miR-125, miR-146, miR-223 and miR-195 enhanced PE/ST-derived cardiomyogenesis, respectively. Importantly, regulation of these microRNAs is distinct modulated by Bmp2 and Fgf2 administration in chicken. In this study, we aim to dissect the functional role of Bmp and Fgf signaling during mouse PE/ST development, their implication regulating post-transcriptional modulators such as microRNAs and their impact on lineage determination. Mouse PE/ST explants and epicardial/endocardial cell cultures were distinctly administrated Bmp and Fgf family members. qPCR analyses of distinct microRNAs, cardiomyogenic, fibrogenic differentiation markers as well as key elements directly epithelial to mesenchymal transition were evaluated. Our data demonstrate that neither Bmp2/Bmp4 nor Fgf2/Fgf8 signaling is capable of inducing cardiomyogenesis, fibrogenesis or inducing EMT in mouse PE/ST explants, yet deregulation of several microRNAs is observed, in contrast to previous findings in chicken PE/ST. RNAseq analyses in mouse PE/ST and embryonic epicardium identified novel Bmp and Fgf family members that might be involved in such cell fate differences, however, their implication on EMT induction and cardiomyogenic and/or fibrogenic differentiation is limited. Thus our data support the notion of species-specific differences regulating PE/ST cardiomyogenic lineage commitment.

## Introduction

Bmp and Fgf signaling are widely involved in multiple aspects of embryonic development (Ornitz and Itoh, 2015; Ornitz and Marie, 2015; Wu et al., 2016; Graf et al., 2016; Salazar et al., 2016; Zinski et al., 2018; Xie et al., 2020; Mossahebi-Mohammadi et al., 2020). Within the developing cardiovascular system, Bmp and Fgf signaling plays essential roles in the determination and specification of the cardiogenic progenitors (Cohen et al., 2007; Hutson et al., 2010; Tirosh-Finkel et al., 2010; Razy-Krajka et al., 2018) as well as in other cardiovascular morphogenetic events such as valve development (Zhao B. et al., 2007; Cushing et al., 2008; Zhang et al., 2010; Zhang et al., 2012). In particular, the role of Bmp and Fgf has also been reported during the formation of the proepicardium/septum transversum (PE/ST) (Torlopp et al., 2010), providing signaling cues to direct the pericardial mesoderm to either proepicardial or myocardial fate (Kruithof et al., 2006; van Wijk et al., 2009). In this context, Bmp2 stimulates cardiomyocyte formation while Fgf2 stimulates epicardial differentiation in chicken embryos. Importantly, there are several discrepancies as whether the PE/ST is capable of giving rise to myocardial cells in mice (Cai et al., 2008; Zhou et al., 2008) as well as whether proepicardial cells are already committed to give rise to distinct cell types such as fibroblasts, endothelial and/or smooth muscle cells at this early developmental stage (Merki et al., 2005; Cai et al., 2008; Zhou et al., 2008; Red-Horse et al., 2010).

Bmp and Fgf signaling have been reported to be modulated by microRNAs (Wang et al., 2010; Icli et al., 2013; Lu et al., 2013; Gan et al., 2016; Pravoverov et al., 2019). In particular, during early cardiac progenitor differentiation, miR-130 has been reported to modulate Fgf8-Bmp2 signaling (Lopez-Sanchez et al., 2015a), providing an intricate regulatory feedback mechanism between with these growth factors and miR-130 that defines the temporal and spatial cues of cardiomyogenic lineage differentiation. More recently we have reported that over-expression of distinct microRNAs in chicken PE/ST, particularly miR-195 and miR-223, influenced cell fate determination of the PE cells, leading to increased formation of myocardial cells, a process that is dependent of Smurf1 and Smad3 (Dueñas et al., 2020). Importantly, miR-195 is distinctly modulated by Bmp and Fgf signaling, supporting a role of this microRNA in the Bmp- and Fgf-directed PE cell specification (Dueñas et al., 2020).

However, scarce evidences are reported as whether Bmp and Fgf signaling plays a role in PE development in mice (Li et al., 2017; Shah et al., 2017). In this context, it is important to highlight that mouse and chicken morphogenesis displayed substantial differences. In mice, two bilateral PE anlage are formed that subsequently fused in the embryonic midline to provide a single PE (Torlopp et al., 2010) while in chicken two anlagen are also formed but only the right-sided one is finally fully developed (Schlueter et al., 2006; Schulte et al., 2007; Schlueter and Brand, 2009; Schlueter and Brand, 2013). While the signalling pathways driving PE development have been substantially characterized (Schlueter et al., 2006; Schulte et al., 2007; Schlueter and Brand, 2009; Schlueter and Brand, 2013), our current understanding of the molecular mechanisms directing murine PE development is still incipient. In addition, epicardial colonization of the embryonic myocardium is also distinctly achieved between chicken and mice (Perez-Pomares et al., 1998; Vrancken Peeters et al., 1999; Manner et al., 2001; Perez-Pomares et al., 2002; Hirose et al., 2006). Furthermore, to date, no evidence of the functional role of discrete microRNAs have been reported regarding the development of the PE in mice, yet deletion of the *Dicer* processing enzyme highlighted their importance (Singh et al., 2011).

In this study, we dissected the functional role of Bmp and Fgf signaling during mouse PE/ST development, their implication regulating post-transcriptional modulators such as microRNAs and their impact on lineage determination. Mouse PE/ST explants, epicardial and endocardial cell cultures were distinctly administrated Bmp and Fgf family members. qPCR analyses demonstrates that Bmp and Fgf family members distinctly regulate microRNAs with potential to inhibit or to enhance PE/ST-derived cardiomyogenic differentiation. Surprisingly, neither those microRNAs with inducing capacity nor the cardiomyogenic inducing capacity previously documented in chicken PE/ST explants was recapitulated in mouse PE/ST explants, supporting the notion of specific-specific differences in PE/ST mouse and chicken development and lineage commitment response.

## Materials and Methods

### Isolation of Proepicardium/Septum Transversum Explants

Experimental protocols were performed in agreement with the Spanish law in application of EU Guidelines for animal research. These protocols conformed to the Guide for Care and Use of Laboratory Animals, published by the US National Institutes of Health (NIH publication no. 85–23). Approved consent of the Ethic Committee of the University of Jaen was obtained prior to the initiation of the study. CD1 pregnant female mice were obtained at embryonic day (ED) 9.5. Embryos were removed from the uterus using iredectomy scissors and placing them into Earle’s balanced salt solution (EBSS) (Gibco). PE/ST were manually dissected and transferred to EBSS solution. Subsequently they were placed into DMEM culture medium, cultured in hanging drops until appropriately treated with different growth factors and/or transfection agents as detailed below.

### EPIC and MEVEC Cell Cultures

Inmortalized embryonic endocardial MEVEC (D’Amato et al., 2016) and epicardial EPIC (Ruiz-Villalba et al., 2013) cells (6 × 10^5^ cells per well) were cultured in DMEM medium supplemented with 10% fetal bovine serum, 100 U/mL penicillin, 100 μg/ml streptomycin and 200 nM of l-glutamine in 100 cm^2^ culture disk at 37°C in a humidified atmosphere of 5% CO_2_, respectively. Cells were fed every 2–3 days. Sub-cultured cells were treated with different growth factors (50 ng/μL) as detailed below for 24 h.

### Growth Factor Administration

PE/ST explants were treated for 24 h with Bmp2, Bmp4, Bmp6, Bmp7, Bmp10, Fgf2, Fgf5, Fgf7, Fgf8 and Fgf10 (Peprotech, East Brunswick, NJ, United States), respectively, as reported by Dueñas et al. (2020). Tissue explants were collected and processed according for qPCR and/or immunohistochemistry. Each experimental condition was carried out in isolated tissues from at least 20 embryos. In all cases, 3–5 independent biological replicates were analyzed.

### MicroRNA Transfections

Mouse E9.5 PE explants were cultured on collagen gels for 24 h at 37°C in a cell culture incubator before pre-miRNAs (microRNA precursors) administration as previously reported (Bonet et al., 2015; Dueñas et al., 2020). Pre-miRNAs (Thermo-Fisher) transfections were carried out with Lipofectamine 2000 (Invitrogen), following the manufacturer’s guidelines. Briefly, 85 nM of pre-miRNA were applied to the explants (3–5 explants per well) for 24 h. After incubation, explants were processed for immunohistochemical (IHC) analyses. Negative controls, i.e. E9.5 PE explants treated only with Lipofectamine, were run in parallel. To perform IHC analyses, the explants were fixed with 1% PFA for 2 h at 4°C, rinsed for three times in PBS during 10 min, and stored in PBS at 4°C. Each experimental condition was carried out in isolated tissues from at least 20 embryos. In all cases, 3–5 independent biological replicates were analyzed.

### Immunofluorescence Analyses by Confocal Laser Scanning Microscopy

Immunofluorescence analyses were performed as previously reported (Bonet et al., 2015; Dueñas et al., 2020). Briefly, control and experimental mouse E9.5 PE explants were collected after the corresponding treatment, rinsed in PBS for 10 min at room temperature, and fixed with 1% PFA for 2 h at 4°C. After fixation, the samples were rinsed three times (10 min each) in PBS at room temperature and then permeabilized with 1% Triton X-100 in PBS for 30 min at room temperature. To block nonspecific binding sites, PBS containing 5% goat serum and 1% bovine serum albumin (Sigma) was applied to the explants overnight at 4°C. As primary antibody, a polyclonal goat anti-cardiac troponin I (Hytest) was used, diluted (1:200) in PBS, and applied to each culture overnight at 4°C. Subsequently, the samples were rinsed three times (for 1 h each) in PBS to remove excess primary antibody and incubated overnight at 4°C with Alexa-Fluor 546 anti-goat (1:100; Invitrogen) as secondary antibody. After incubation with the secondary antibody, the explants were rinsed as described above. Finally, the explants and/or epicardial cell cultures, respectively, were incubated with phalloidin (1:1,000; Thermo-Fisher) overnight, and DAPI (1:1,000; Sigma) for 7 min at room temperature and rinsed three times in PBS for 5 min each. Explants were stored in PBS in darkness at 4°C until analyzed using a Leica TCS SP5 II confocal scanning laser microscope.

### RNA Isolation and qPCR Analyses

All RT-qPCR experiments followed MIQE guidelines (Bustin et al., 2001) and similarly as previously reported (Bonet et al., 2015; Lozano-Velasco et al., 2015; Dueñas et al., 2020). Briefly, RNA was extracted and purified by using Trizol reactive (Invitrogen) according to the manufacturer’s instructions. For mRNA expression measurements, 1 μg of total RNA was used for retro-transcription with Maxima First Strand cDNA Synthesis Kit for RT-qPCR (Thermo Scientific). Real time PCR experiments were performed with 1 μL of cDNA, SsoFast EvaGreen mix and corresponding primer sets. For microRNA expression analyses, 20 ng of total RNA was used for retrotranscription with Universal cDNA Synthesis Kit II (Qiagen) and the resulting cDNA was diluted 1/80. Real time PCR experiments were performed with 1 μL of diluted cDNA, ExiLENT SYBR Green master mix (Qiagen) and corresponding primer sets. microRNA primers were purchased from ThermoFisher and mRNA primers were custom designed using Primer3 software (Supplementary Table S1). All qPCRs were performed using a CFX384TM thermocycler (Bio-Rad) following the manufacturer’s recommendations. The relative level of expression of each gene was calculated as described by Livak and Schmittgen (2001) using *Gapdh* and *Gusb* as internal control for mRNA expression analyses and *5S* and *6U* for microRNA expression analyses, respectively. Each PCR reaction was carried out in triplicate and repeated in at least three distinct biological samples to obtain representative means.

### Statistical Analyses

For statistical analyses of datasets, unpaired Student’s t-tests were used, as previously reported (Bonet et al., 2015; Dueñas et al., 2020). Significance levels or *p* values are stated in each corresponding figure legend. *p* < 0.05 was considered statistically significant.

### Mouse Lines and Tissue Collection

Previously described Wt1^GFP/+^ mice used in this study (Cano et al. 2013). Pregnant Wt1^GFP/+^ female mice were harvested to E9.5 and to E10.5, respectively. E9.5 PE were manually dissected, pooled and stored in liquid nitrogen until used. E10.5 EE was FACS-sorted as previously described, pooled and stored in liquid nitrogen until used. At least 3-5 litters were used on each developmental stage until sufficient tissues was collected that would guarantee optimal RNA isolation.

### miRNAseq Library Preparation, Sequencing and Proccesing of FastQ Files

500 pg of total RNA were used to generate barcoded miRNA-seq libraries using the *Bioo NEXTflex Small RNA* (BiooScientific). Briefly, 3′ and 5′ SR adapters were first ligated to the RNA sample. Next, reverse transcription followed by PCR amplification was used to enrich cDNA fragments with adapters at both ends. Adapter-ligated cDNA fragments from different samples were pooled and run in a 6% polyacrilamide gel. The 147 nt band, corresponding to the pooled miRNA libraries, was purified from the gel. Finally, the quantity and quality of the pooled miRNA libraries were determined using the Agilent 2100 Bioanalyzer High Sensitivity DNA chip. Libraries were sequenced on a HiSeq 2500 (Illumina) and processed with RTA v1.18.66.3. FastQ files for each sample were obtained using bcl2fastq v2.20.0.422 software (Illumina). Sequencing reads were aligned to the mouse reference genome (mm10) with HISAT2 v2.10.0 (Kim et al., 2015) and then extracted the miRNA counts with featureCounts (Liao et al., 2014) and miRBase (Kozomara and Griffiths-Jones 2014) GFF3 for mouse. Raw counts were normalized with TPM (Transcripts per Million) and TMM (Trimmed Mean of M-values) methods, transformed into log2 expression [log2 (rawCount+1)] and compared to calculate fold-change and corrected pValue. The limits for the differential expression were Log2FC > 0.584 (1.5x) and corrected p Value <0.05. Only miRNAs detected in the three replicates of any condition were use in the analysis. These data are uploaded into Gene Expresssion Onmibus platform with accession number GSE189344.

### mRNAseq Library Preparation, Sequencing and Proccesing of FastQ Files

First 2.5 ng of total RNA were used to amplify the cDNA using the *SMART-Seq v4 Ultra Low Input RNA Kit* (Clontech-Takara). 1 ng of amplified cDNA was used to generate barcoded libraries using the *Nextera XT DNA library preparation kit* (Illumina). Basically, cDNA is fragmented and adapters are added in a single reaction followed by an amplification and clean up. The size of the libraries was checked using the Agilent 2100 Bioanalyzer High Sensitivity DNA chip and their concentration was determined using the Qubit® fluorometer (ThermoFisher Scientific). Libraries were sequenced on a HiSeq 2500 (Illumina) and processed with RTA v1.18.66.3. FastQ files for each sample were obtained using bcl2fastq v2.20.0.422 software (Illumina). Sequencing reads were aligned to the mouse reference transcriptome (mm10 v92) and quantified with RSem v1.3.1 (Li and Dewey 2011). Raw counts were normalized with TPM (Transcripts per Million) and TMM (Trimmed Mean of M-values) methods, transformed into log2 expression [log2 (rawCount+1)] and compared to calculate fold-change and corrected pValue. The limits for the differential expression were Log2FC > 0.584 (1.5x) and corrected pValue <0.05. Only mRNAs detected in three transcriptomes were use in the analysis. These data are uploaded into Gene Expresssion Onmibus platform with accession number GSE189344.

### Heatmap Representation

Normalized RNAseq data were graphically plotted as heatmaps using Morpheus software (https://software.broadinstitute.org/morpheus/).

## Results

### miR-195 and miR-223 Does Not Enhance Proepicardium-Derived Cardiogenesis in Mice

**FIGURE 1:**
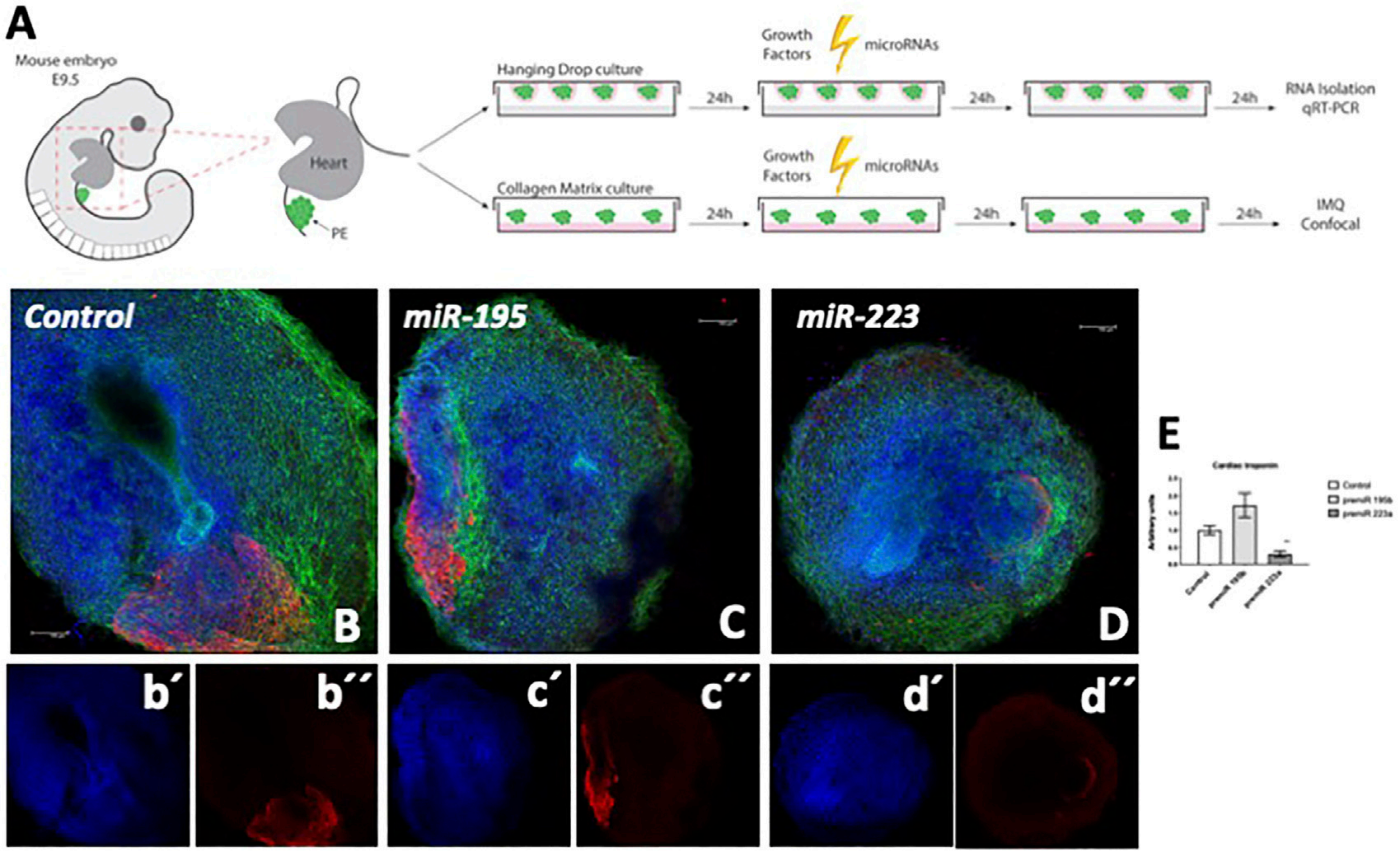
Panel **(A)** Schematic representation of the experimental design of mouse PE/ST isolation, transfection with microRNAs or growth factor administration and subsequent analyses. Panels **(B–D)** Confocal microscopy analyses of cardiac troponin expression (red) in mouse E9.5 PE/ST explants treated with control **(B)** miR-195 mimics **(C)**, miR-223 mimics (D), respectively. Single channel confocal images of Dapi and cardiac troponin are depicted for control (b´, b´´), miR-195 (c´, c´´) and miR-233 (d´, d´´) treated explants, respectively. Panel **(E)** displays quantitation of mean cardiac troponin positive areas in each experimental condition (*n* = 5). Note that the cardiac troponin expression is similarly observed in miR-195 treated explants and controls, while a significant decreased in miR-223 treated explants is observed. Blue (DAPI), phalloidin (green).

Previous reports in our laboratory demonstrated that ectopic administration of miR-195 and miR-223, respectively, enhanced cardiomyogenesis in PE/ST explants in chicken (Dueñas et al., 2020). We now tested whether this process is also occurring in mouse PE/ST explants. As depicted in Figure 1A, mouse PE/ST explants were dissected and treated with miR-195 and miR-223, respectively, for 24 h and subsequently fixed for confocal image analyses. Analyses of cardiomyocyte terminal differentiation marker cardiac troponin demonstrate that neither miR-195 nor miR-223 enhanced cardiomyogenesis in mouse PE/ST explants (Figures 1B–E).

### Bmp and Fgf Signalling in Mouse PE E9.5 Explants

**FIGURE 2:**
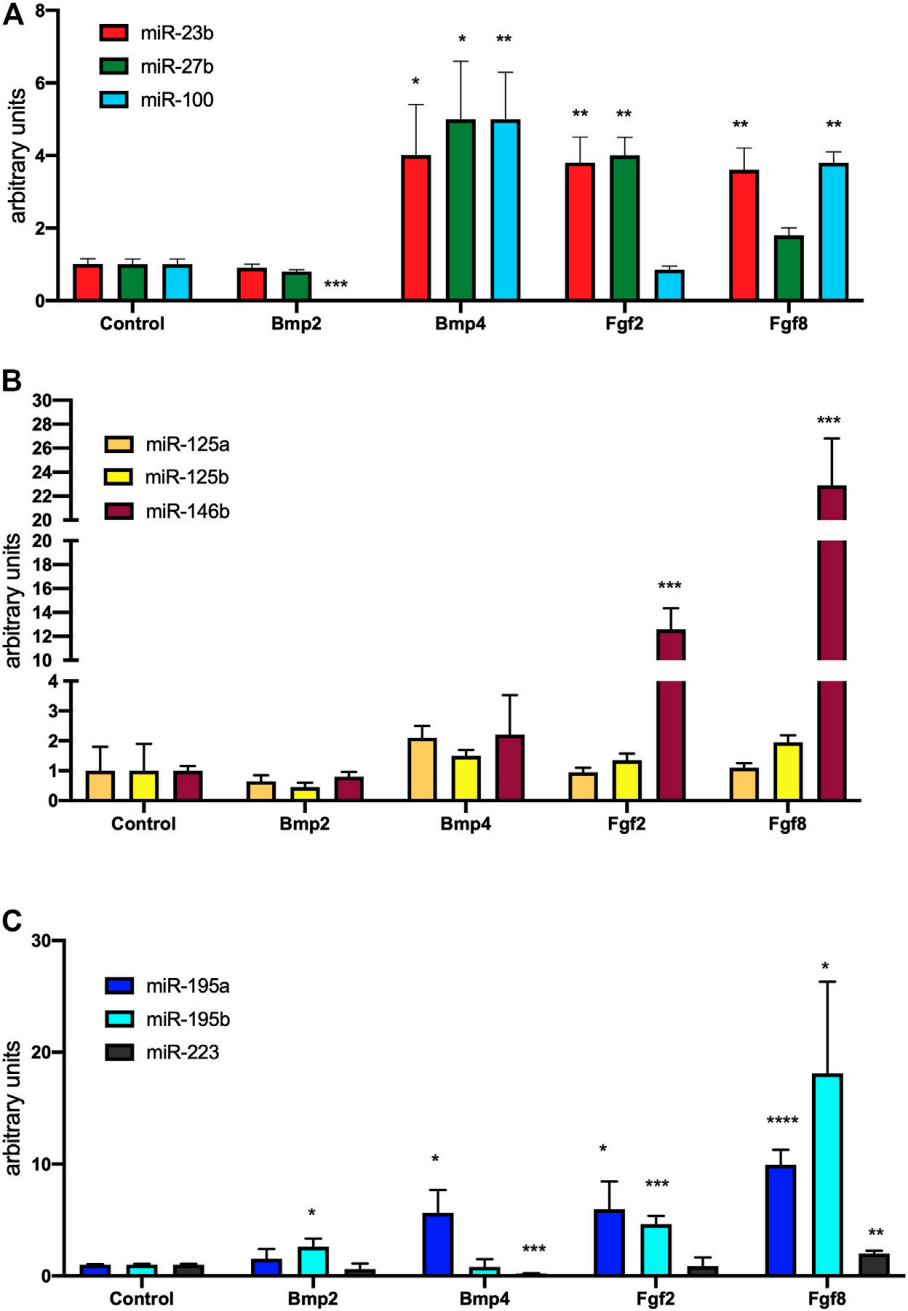
Panel **(A)** RT-qPCR analyses of miR-23b, miR-27b and miR-100 expression after control, Bmp2, Bmp4, Fgf2 and Fgf8 treatments, respectively, to mouse E9.5 PE/ST explants. Panel **(B)** RT-qPCR analyses of miR-125a, miR-125b and miR-146b expression after control, Bmp2, Bmp4, Fgf2 and Fgf8 treatments, respectively, to mouse E9.5 PE/ST explants. Panel **(C)** RT-qPCR analyses of miR-195a, miR-195b and miR-223 expression after control, Bmp2, Bmp4, Fgf2 and Fgf8 treatments, respectively, to mouse E9.5 PE/ST explants. *p < 0.05, **p < 0.01, ***p < 0.001, ****p < 0.0001.

We have previously reported that Bmp and Fgf family members, can distinctly modulate the expression of microRNAs that can differently modulate PE/ST-derived cardiomyogenesis. Thus, we have administered Bmp2, Bmp4, Fgf2 and Fgf8 to mouse E9.5 PE explants and tested the expression of distinct microRNAs previously involved in PE cell determination in chicken. Bmp2 administration eliminated miR-100 expression, increased miR-195b expression while miR-23b, miR-27b, miR-125a, miR-125b, miR-146b, miR-195a and miR-223 displayed no significant differences (Figure 2). Bmp4 administration increased miR-23b, miR-27b, miR-100 and miR-195a, eliminated miR-223 while no significant differences were observed for miR125a, miR-125b and miR-195b (Figure 2). Of note, miR-125a miR-125b and miR-146b display an enhanced trend but reached no statistical significance. Fgf2 administration increased miR-23b, miR-27b, miR-146b, miR-195a and miR-195b, while no changes were observed for miR-100, miR-125a, miR-125b and miR-223 (Figure 1). Finally, Fgf8 administration leads to increase of miR-23b, miR-100, miR-146b, miR-195a, miR-195b and miR-223, but no significant differences on miR-27b, miR-125a and miR-125b expression (Figure 2). Overall, these data demonstrate that distinct Bmp and Fgf family members can differentially modulate those microRNAs that significantly enhanced cardiomyogenesis (miR-195a, miR-195b and miR-223), those that mildly enhanced it (miR-125a, miR-125b and miR-146b) and those that do not enhance or even inhibit it (miR-23b, miR-27b and miR-100). Surprisingly, enhanced miR-195a, miR-195b and/or miR-223 is similarly observed for Bmp2/Bmp4 *vs* Fgf2/Fgf8 in mouse PE/ST explants, in contrast to our previous findings in chicken PE/ST, suggesting clear species-specific differences.

**FIGURE 3:**
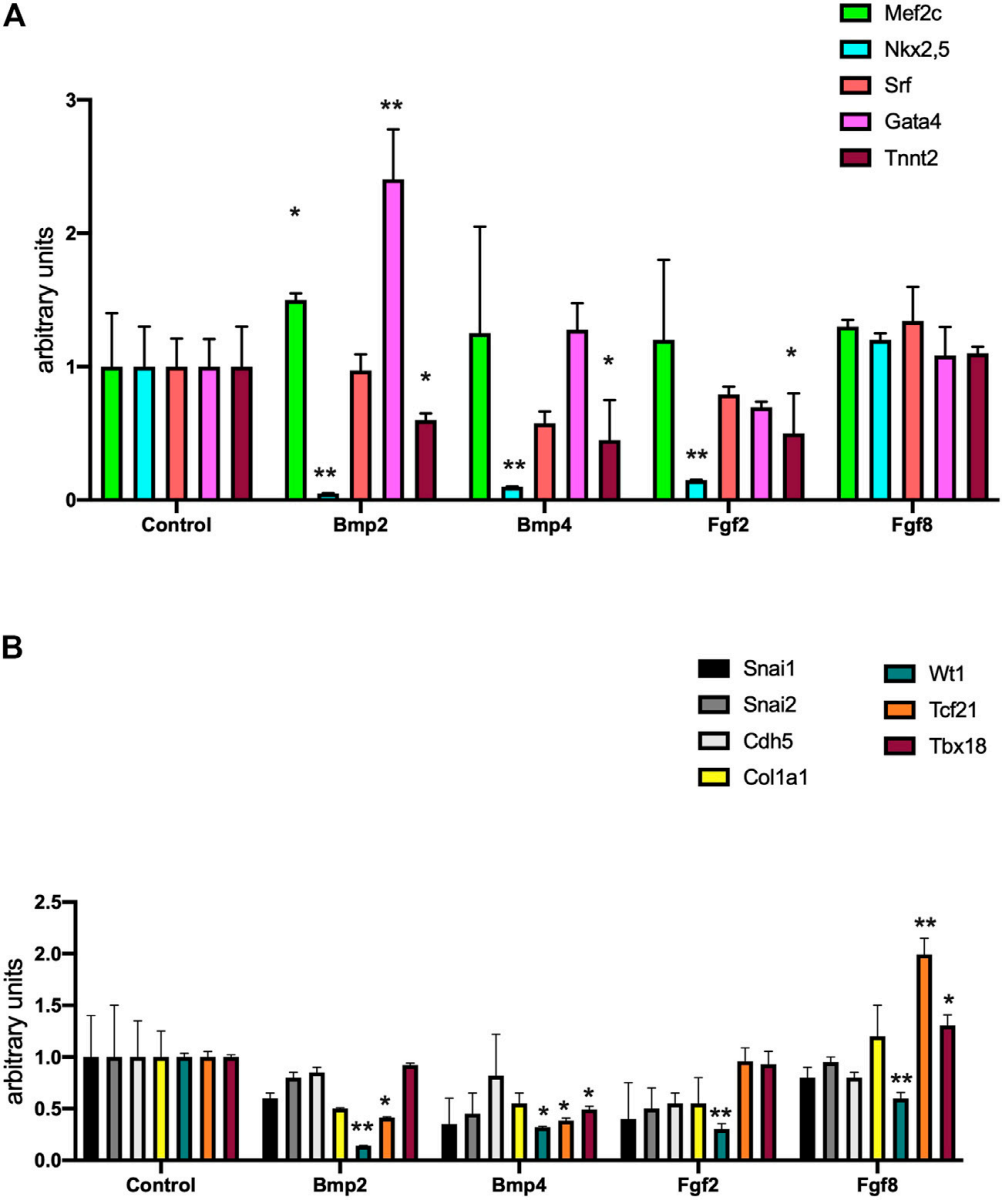
Panel **(A)** RT-qPCR analyses of Mef2c, Nkx2.5, Srf, Gata4 and Tnnt2 expression after control, Bmp2, Bmp4, Fgf2 and Fgf8 treatments, respectively, to mouse E9.5 PE/ST explants. Panel **(B)** RT-qPCR analyses of Snai1, Sna2, Cdh5, Col1a1, Wt1, Tcf21 and Tbx18 expression after control, Bmp2, Bmp4, Fgf2 and Fgf8 treatments, respectively, to mouse E9.5 PE/ST explants. *p < 0.05, **p < 0.01, ***p < 0.001, ****p < 0.0001.

We subsequently tested if these growth factors could influence the expression of molecular markers involved in early (Mef2c, Nkx2.5, Gata4, Srf) and terminal (Tnnt2) differentiation of cardiomyogenesis. Bmp2 administration significantly increased Mef2c and Gata4 expression, Nkx2.5 and Tnnt2 were decreased and Srf displayed no significant differences (Figure 3A). Bmp4 and Fgf2 administration decreased Nkx2.5 and Tnnt2 while no significant differences were observed for the other markers analyzed (Figure 3A). Fgf8 administration did not modify any of the markers analyzed (Figure 3A). Importantly, analyses of fibrogenesis (Col1a1) and epithelial to mesenchymal transition (EMT) (Snail1, Snail2, Cdh5) markers were not significantly modulated by any of these growth factors, i.e. Bmp2, Bmp4, Fgf2, and Fgf8 (Figure 3B) while epicardial markers such as Wt1, Tcf21 and Tbx18 were either inhibited and/or not modified. Only Fgf8 administration significantly increased Tcf21 and Tbx18 expression (Figure 3B).

### Differential Expression of Bmp and Fgf Family Members in Mouse PE/EE Transition

**FIGURE 4:**
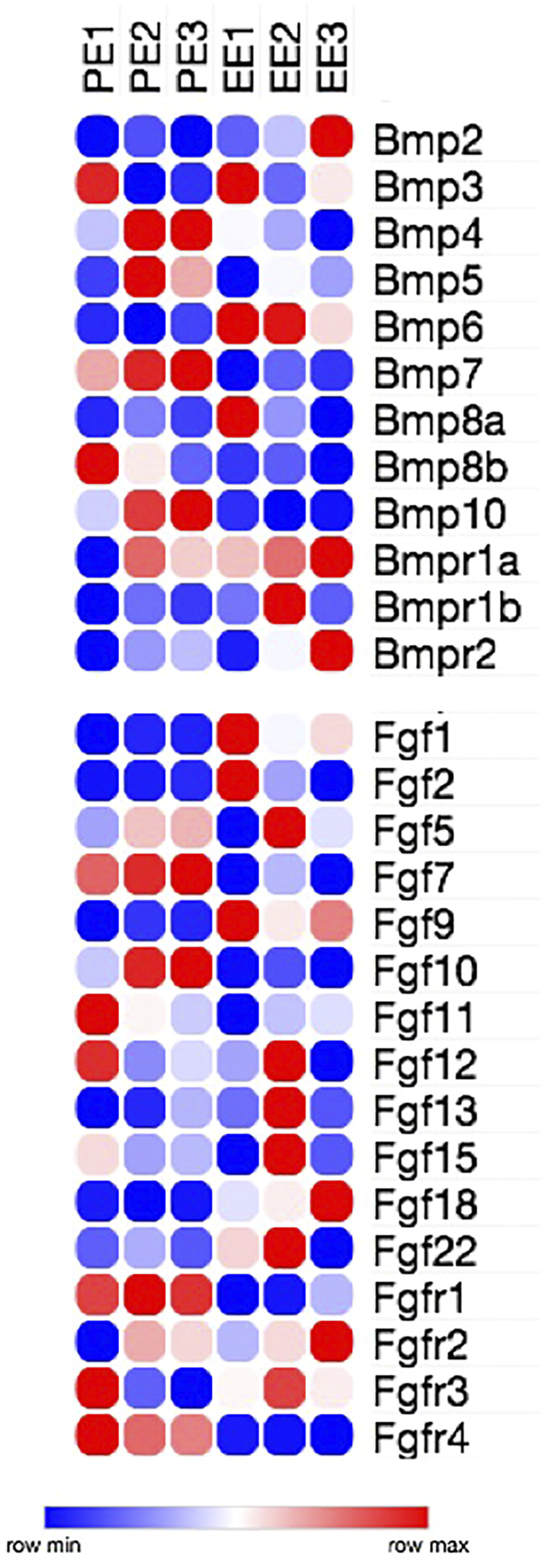
Heatmap representation of Bmp and Fgf family members expression as revealed by RNAseq analyses in mouse E9.5 PE (PE1, PE2 and PE3) and E10.5 embryonic epicardium (EE1, EE2 and EE3).

Given the fact that Bmp2, Bmp4, Fgf2 and Fgf8 distinct modulate microRNA expression and cardiomyogenic lineage determination in chicken and mouse PE/ST, and given the divergent morphogenetic events that occur between these two species, we took advantage of our recent performed comprehensive analysis of coding and non-coding RNA differential gene expression in mouse E9.5 proepicardium vs E10.5 embryonic epicardium to unravel the gene regulatory networks involved in PE to EE transition to search for novel Bmp and Fgf members that might be involved in this process (Franco et al., in preparation). Importantly, differential expression of growth factors during these developmental conditions have unraveled Bmp4, Bmp5, Bmp7, and Bmp10 are highly expressed in the PE at E9.5 while Bmp2, Bmp3 and Bmp6 display enhanced expression at E10.5 embryonic epicardium (Figure 4). In line with previous reports in chicken PE development, Bmp2 and Bmp4 are distinctly expressed during PE/EE development, but in addition novel Bmp members are also identified during mouse PE that were unnoticed during chicken development, such as Bmp5, Bmp7 and Bmp10 that might plays significant roles during PE/EE transition. Similarly, Fgf5, Fgf7, Fgf10, Fgf11 and Fgf12 are highly expressed in the PE at E9.5 while Fgf1, Fgf2, Fgf5, Fgf9 and Fgf18 display enhanced expression at E10.5 embryonic epicardium (Figure 4). Surprisingly, Fgf8 was not detected in our RNAseq analyses, pointing out to differential Fgf expression during PE/EE development in chicken and mice. Furthermore, our data also unraveled novel Fgf members that might be potentially involved in mouse PE/EE development, such as Fgf5, Fgf7, Fgf10, Fgf11 and Fgf12.

### Novel Regulatory Roles of Bmp and Fgf Family Members During PE/ST Differentiation

**FIGURE 5:**
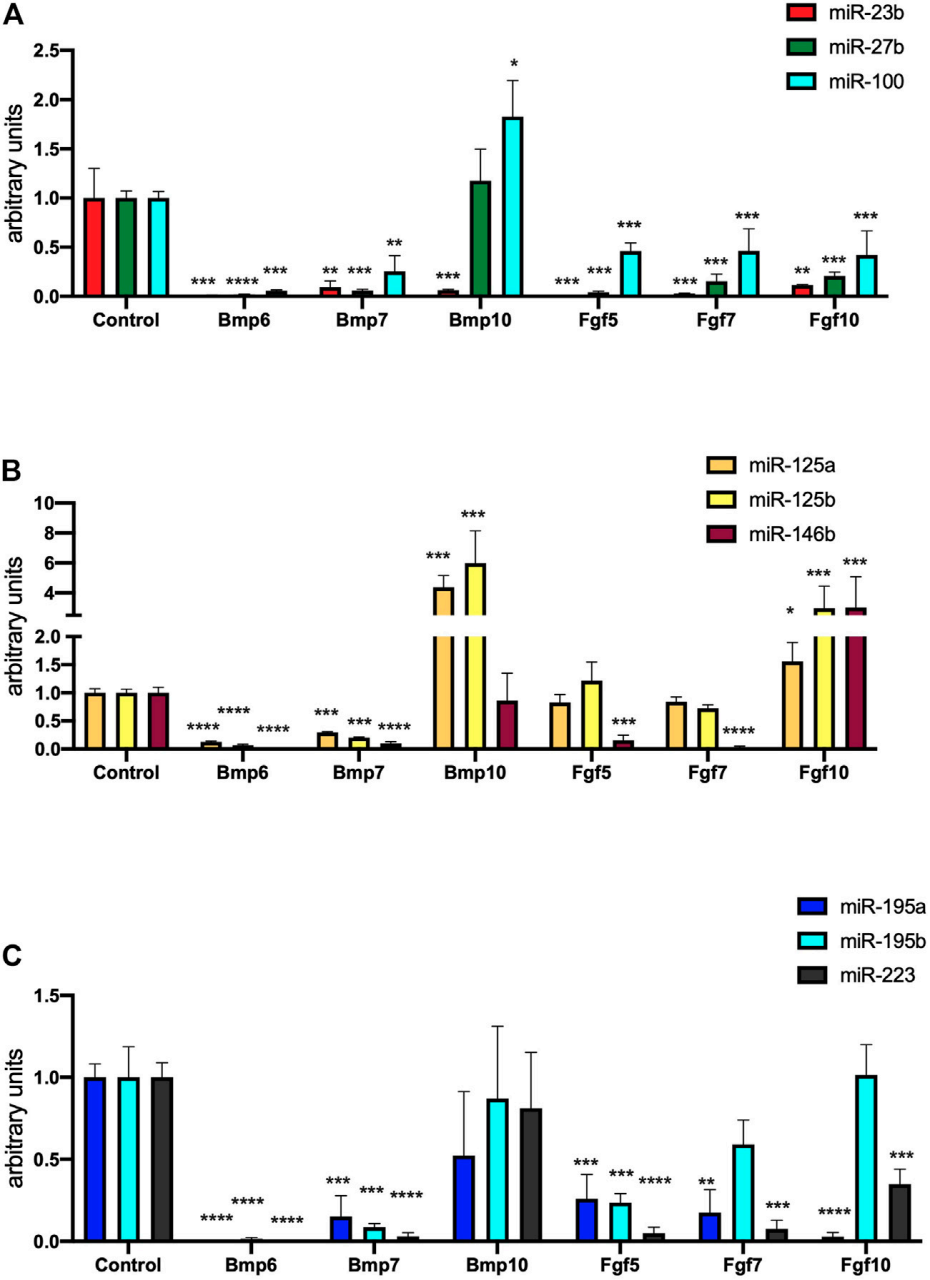
Panel **(A)** RT-qPCR analyses of miR-23b, miR-27b and miR-100 expression after control, Bmp6, Bmp7, Bmp10, Fgf5, Fgf7 and Fgf10 treatments, respectively, to mouse E9.5 PE/ST explants. Panel **(B)** RT-qPCR analyses of miR-125a, miR-125b and miR-146b expression after control, Bmp6, Bmp7, Bmp10, Fgf5, Fgf7 and Fgf10 treatments, respectively, to mouse E9.5 PE/ST explants. Panel **(C)** RT-qPCR analyses of miR-195a, miR-195b and miR-223 expression after control, Bmp6, Bmp7, Bmp10, Fgf5, Fgf7 and Fgf10 treatments, respectively, to mouse E9.5 PE/ST explants. *p < 0.05, **p < 0.01, ***p < 0.001, ****p < 0.0001.

To dissect the plausible signaling role these differentially expressed Bmp and Fgf family members during mouse PE/ST development, we administered Bmp6, Bmp7, Bmp10, Fgf5, Fgf7 and Fgf10 to mouse PE/ST explants and analyzed their role in microRNA and lineage specific expression. Bmp6 and Bmp7 administrated blunted expression of all microRNAs analyzed (miR-23b, miR-27b, miR-100, miR-125a, miR-125b, miR-146b, miR-195a, miR-195b and miR-223) (Figure 5). On the other hand, Bmp10 overexpression significantly enhanced miR-27b, miR-100, miR-125a, decreased miR-23b and while no differences were observed for the rest of microRNAs analyzed (miR-146b, miR-195a, miR-195b and miR-223) (Figure 5). Curiously, Fgf5 and Fgf7 administration also significantly decreased most of the microRNAs studied (miR-23b, miR-27b, miR-100, miR-146b, miR-195a, miR-195b and miR-223), while no significant differences were observed for miR-125a and miR-125b (Figure 5). On the other hand, Fgf10 administration, significantly decreased miR-23b, miR-27b, miR-100, miR-195a and miR-223, significantly enhanced miR-125a, miR-125b and miR-146b and displayed no significant differences for miR-195b (Figure 5). Overall, all these data demonstrate that only Fgf10 and Bmp10 are capable of significantly modulate microRNA expression in mouse PE/ST explants. Surprisingly, none of them is nonetheless capable of enhancing those microRNAs robustly enhance cardiomyogenesis (i.e. miR-195a, miR-195b and miR-223), but they are capable of modulating those that mildly enhance cardiomyogenesis (i.e., miR-125a and miR-125b). Importantly, Bmp10, but not Fgf10 also enhance microRNA expression that leads to consistent cardiomyogenic blockage (miR-27b, miR-100).

**FIGURE 6:**
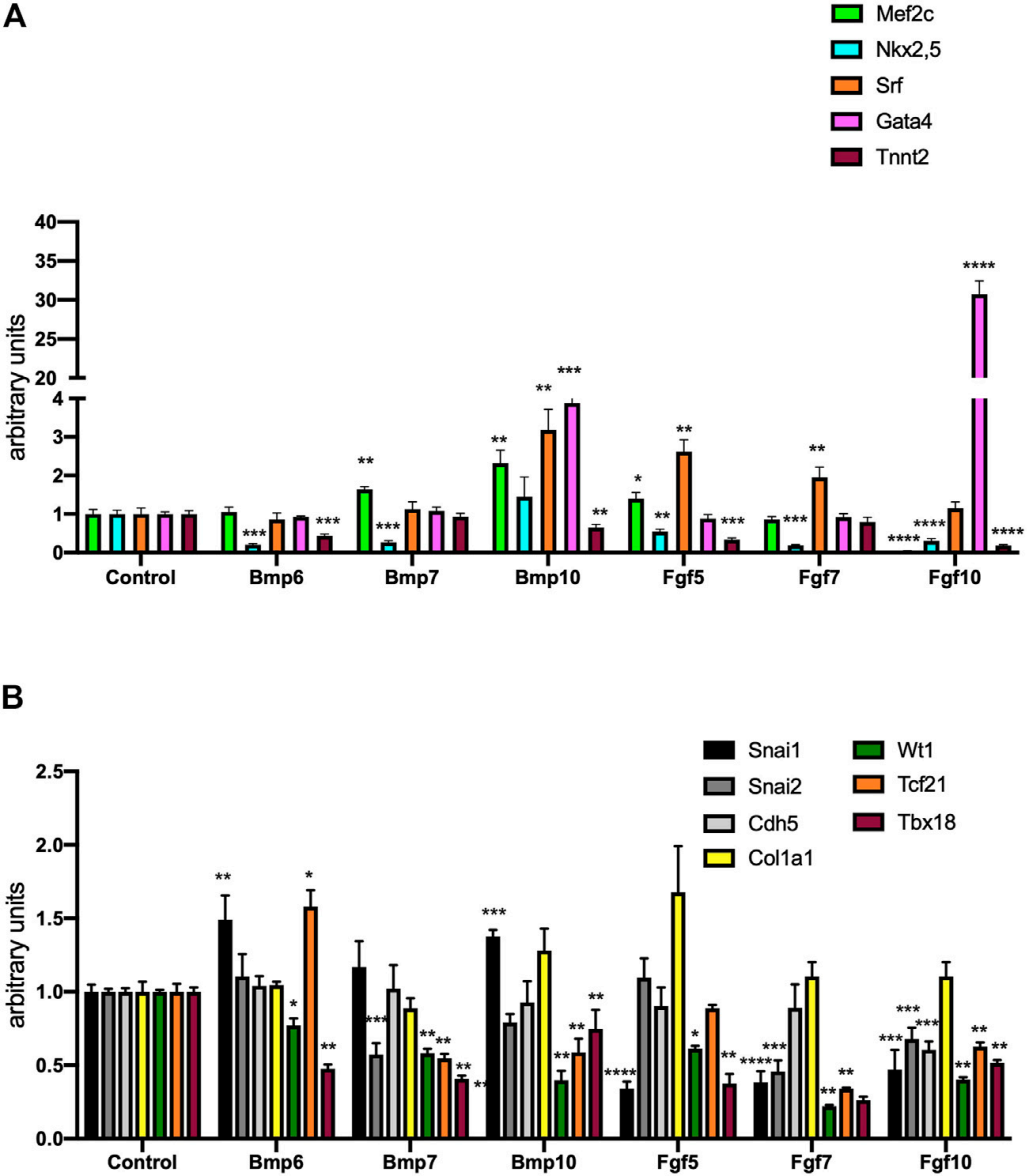
Panel **(A)** RT-qPCR analyses of Mef2c, Nkx2.5, Srf, Gata4 and Tnnt2 expression after control, Bmp6, Bmp7, Bmp10, Fgf5, Fgf7 and Fgf10 treatments, respectively, to mouse E9.5 PE/ST explants. Panel **(B)** RT-qPCR analyses of Snai1, Sna2, Cdh5, Col1a1, Wt1, Tcf21 and Tbx18 expression after control, Bmp6, Bmp7, Bmp10, Fgf5, Fgf7 and Fgf10 treatments, respectively, to mouse E9.5 PE/ST explants. *p < 0.05, **p < 0.01, ***p < 0.001, ****p < 0.0001.

We subsequently tested whether these growth factors can influence cardiomyogenic lineage determination. Bmp6 and Bmp7 significantly decreased Nkx2.5 expression while *Mef2c, Srf* and Gata4 were not significantly decreased. *Tnnt2*, a marker of cardiac terminal differentiation was significantly decreased by Bmp6 but not by Bmp7 administration (Figure 6A). Bmp10 enhanced expression of all cardiomyogenic markers except *Nkx2.5* and *Tnnt2*. Fgf5 and Fgf7 exclusively increased *Srf* expression while the other tested markers were either decreased (Nkx2.5) or not significantly altered (Mef2c, Gata4 and Tnnt2) (Figure 6A). Fgf10 administration resulted in downregulation of Mef2c, Nkx2.5 and Tnnt2, while only Gata4 was up-regulated and Srf displayed no significant differences (Figure 6A). Overall, these data demonstrate that although several of these growth factors can promote upregulation of several early molecular markers of cardiogenesis, none of them is capable of inducing terminal cardiomyocyte differentiation.

**FIGURE 7:**
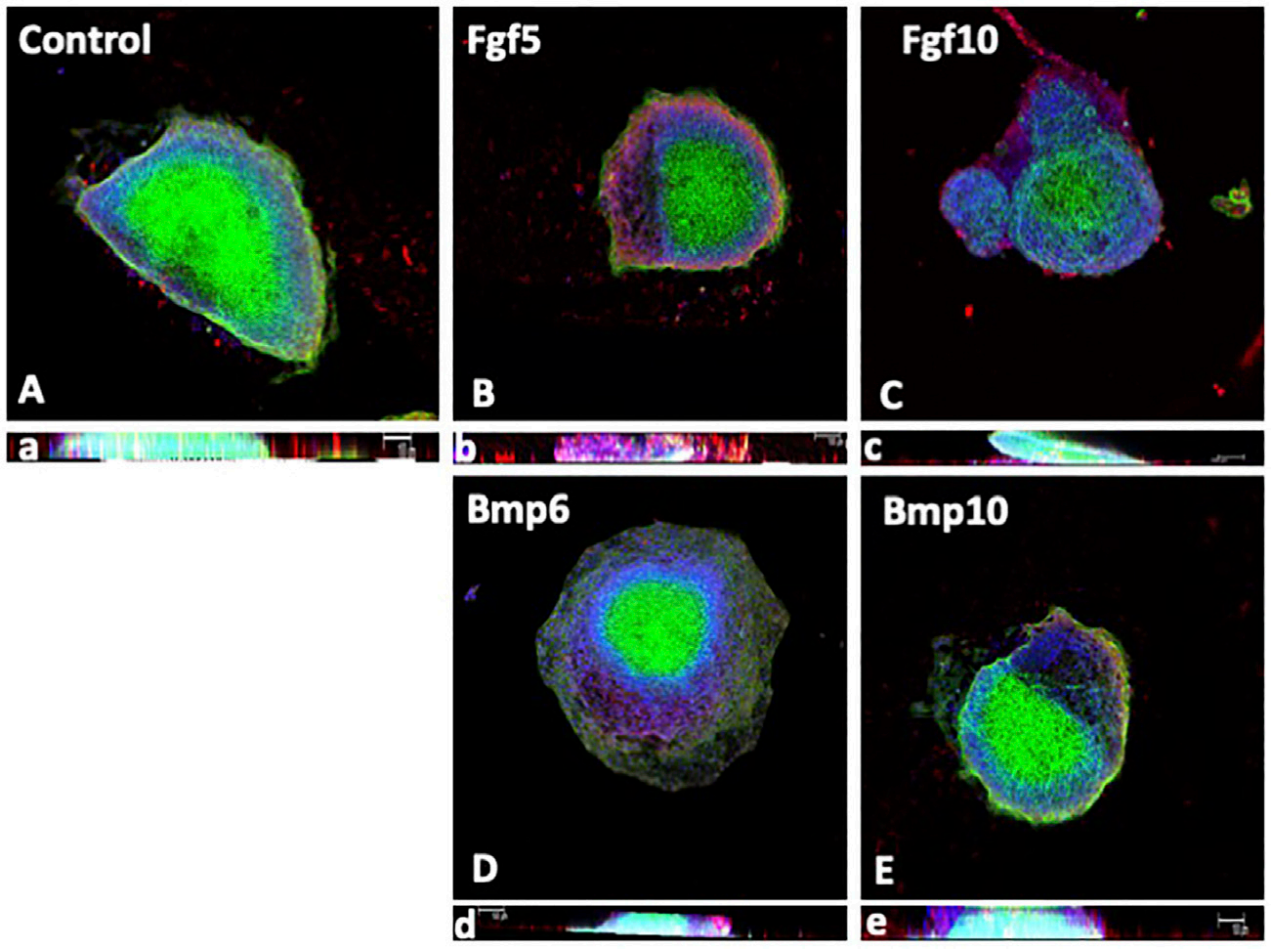
Panels **(A–E)** Confocal images of mouse E9.5 PE/ST explants after control **(A)**, Fgf5 **(B)**, Fgf10 **(C)**, Bmp6 **(D)** and Bmp10 **(E)** treatments, respectively. Note that treatment with Fgf5, Fgf10 and Bmp10 displays limited migration, similar to controls, while Bmp6 is significantly increased. Panels a–e represent Z-stack confocal views, respectively, where it can be observed that none of them display signs of EMT.

We subsequently tested these growth factors can influence EMT and/or fibrogenesis. Bmp6 administration only enhance *Snai1* expression, while *Snai2*, Cdh5 and Col1a1 were not significantly altered (Figure 6B). Bmp7 only decreased Snai2, while Bmp10 only upregulated Snail1 and Col1a1. On the other hand, Fgf5, Fgf7 and Fgf10 significantly down-regulated Snail1, while Snail2 was also downregulated by Fgf7 and Fg10 administration **(** Figure 6B). In addition, Fgf5 upregulated Col1a1 and Fgf10 decreased Cdh5 expression (Figure 6B). Confocal imaging of mouse proepicardial explants treated with Fgf5, Fgf10, Bmp6 and Bmp10, respectively, demonstrate that such growth factors does not significantly promote EMT, in line with our qPCR data (Figure 7). In addition, we also demonstrated that epicardial markers such as Wt1, Tcf21 and Tbx18 were significantly down-regulated, except for Bmp6 administration that significantly increased Tcf21 expression (Figure 6B)**.** In summary, Bmp6 and Bmp10 might mildly enhanced EMT markers but Fgf5, Fgf7 and Fgf10 robustly inhibited them, while none of them effectively promote EMT in explant cultures. Furthermore, fibrogenesis was only up-regulated by Bmp10 and Fgf5.

### Bmp and Fgf Signalling in Epicardial vs. Endocardial Cell Lineages

**FIGURE 8:**
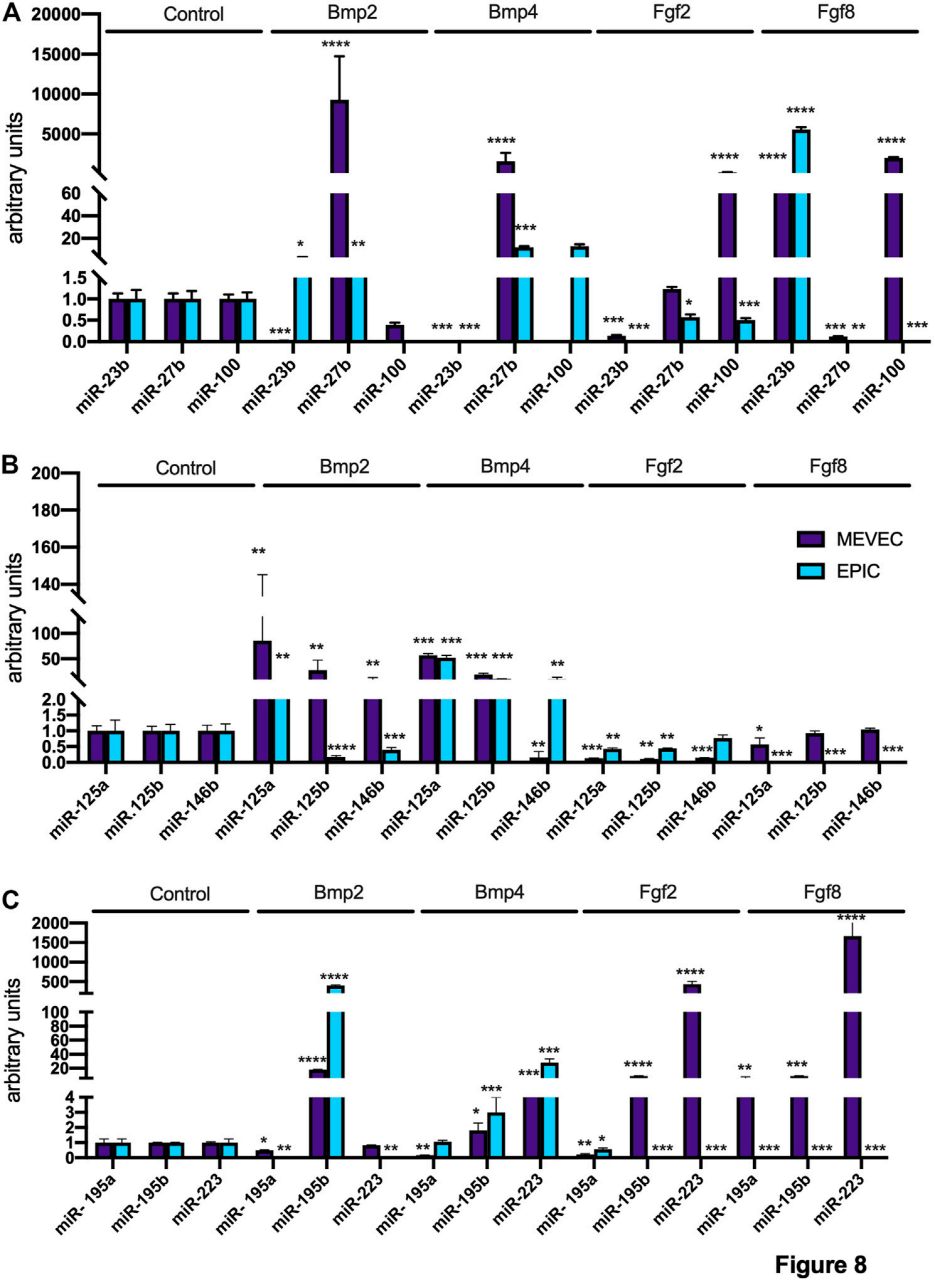
Panel **(A)** RT-qPCR analyses of miR-23b, miR-27b and miR-100 expression after control, Bmp2, Bmp4, Fgf2 and Fgf8 treatments in MEVEC (endocardial) and EPIC (epicardial) cell lines, respectively. Panel **(B)** RT-qPCR analyses of miR-125a, miR-125b and miR-146b expression after control, Bmp2, Bmp4, Fgf2 and Fgf8 treatments in MEVEC (endocardial) and EPIC (epicardial) cell lines, respectively. Panel **(C)** RT-qPCR analyses of miR-195a, miR-195b and miR-223 expression after control, Bmp2, Bmp4, Fgf2 and Fgf8 treatments in MEVEC (endocardial) and EPIC (epicardial) cell lines, respectively. *p < 0.05, **p < 0.01, ***p < 0.001, ****p < 0.0001.

To further support the findings observed in *ex vivo* mouse PE/ST explants, we have administered Bmp2, Bmp4, Fgf2 and Fgf8 to two distinct cell lines, representing epicardial (EPIC; Ruiz-Villalba et al., 2013) and endocardial (MEVEC) cells and tested whether distinct microRNAs previously involved in PE cell determination are modulated by these growth factors. Bmp2 administration enhanced miR-27, miR-125a and miR-195b expression in both cell types, while display opposite regulation in endocardial vs epicardial cells for miR-23b, miR-125b, miR-146b, miR-195a and miR-223 (Figure 8). Bmp4 administration increased miR-27b, miR-125a, miR-125b, miR-195b, miR-223, decreased miR-23b while displayed opposite patterns of regulation for miR-146b, respectively (Figure 8). Fgf2 administration decreased miR-23b, miR-125a, miR-125b and miR-195a while displayed opposite patterns of regulation for miR-100, miR-185b and miR-223 in both cell types, respectively (Figure 8). Fgf8 increased miR-23b, decreased miR-27b and miR-125a in both cell types, while displayed opposite pattern for miR-100, miR-195a, miR-195b and miR-223 (Figure 8).

We subsequently monitored if these growth factors could influence the expression of molecular markers involved in early (Mef2c, Gata4, Srf, Nkx2.5) and terminal (Tnnt2) differentiation of cardiomyogenesis, fibrogenesis (Col1a1) and epithelial to mesenchymal transition (EMT) (Snai1, Snai2, Cdh5).

**FIGURE 9:**
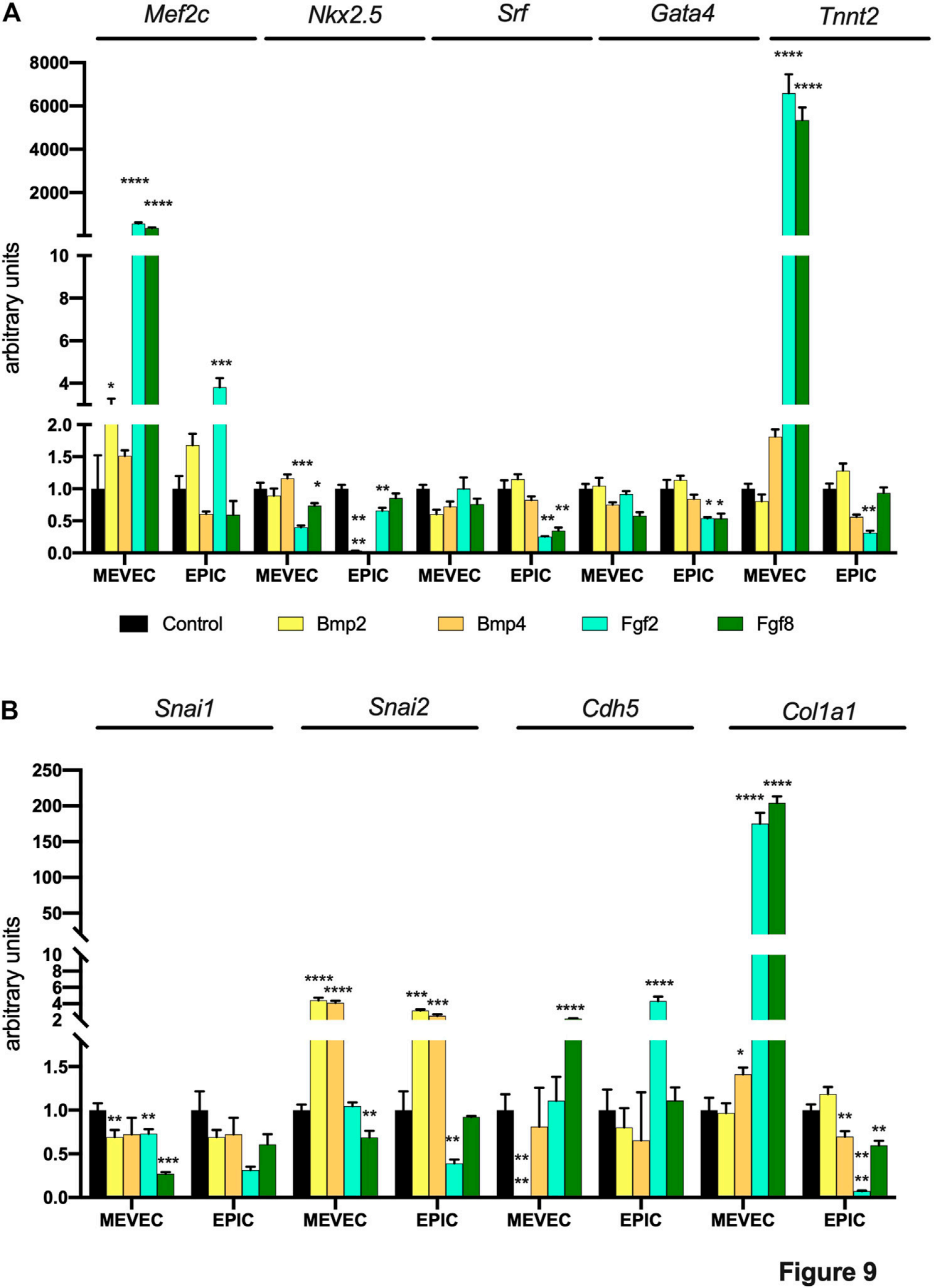
Panel **(A)** RT-qPCR analyses of Mef2c, Nkx2.5, Srf, Gata4 and Tnnt2 expression after control, Bmp2, Bmp4, Fgf2 and Fgf8 treatments in MEVEC (endocardial) and EPIC (epicardial) cell lines, respectively. Panel **(B)** RT-qPCR analyses of Snai1, Snai2, Cdh5 and Col1a1 expression after control, Bmp2, Bmp4, Fgf2 and Fgf8 treatments in MEVEC (endocardial) and EPIC (epicardial) cell lines, respectively. *p < 0.05, **p < 0.01, ***p < 0.001, ****p < 0.0001.

Bmp2 and Bmp4 administration did not modify the expression of any early and terminal differentiation markers in any of the 2 cell types analyzed, except for a significant downregulation of Nkx2.5 in EPIC cells **(** Figure 9). On the other hand, Fgf2 and Fgf8 significantly upregulated Mef2c and Tnnt2 expression in MEVEC, while only Fgf2 administration increased Mef2c but not Tnnt2 expression in EPIC cells (Figure 9). Furthermore, both growth factors, i.e. Fgf2 and Fgf8, decreased Nkx2.5 expression in MEVECs and Srf in EPIC while only Fgf2 administration resulted in down-regulation of Nkx2.5 and Tnnt2 in EPIC cells (Figure 9). Overall, these data demonstrate that only Fgf2 and Fgf8 are inducing terminal cardiomyocyte differentiation in MEVEC but not in EPIC cells.

Analyses of EMT inductors demonstrated that Bmp2 and Bmp4 can enhance the expression of Snai1 in MEVEC cells and Snai2 in EPIC cells, while Cdh5 expression is only downregulated in MEVEC cells by Bmp2 expression (Figure 9). On the other hand, Fgf2 and Fgf8 administration do not modify, or if any decreased, the expression of Snai1 and Snai2 in both cell types. Cdh5 expression is up-regulated by Fgf8 in MEVEC and by Fgf2 in EPIC cells. Fibrogenic marker Col1a1 is significantly upregulated in MEVEC but not in EPIC cells by Fgf2 and Fgf8, while Bmp2 and Bmp4 administrations does not significantly alter its expression in any of the 2 cell types analyzed (Figure 9).

Overall, these data illustrate that these growth factors can distinctly modulate the expression of different microRNAs, previously reported to inhibit (miR-23b, miR-27b and miR-100), to mildly promote (miR-125a, miR-125b, miR-146b) or to substantially enhance (miR-195a, miR-195b, miR-223) cardiomyogenesis in chicken PE/ST. Surprisingly, none of tested growth factor distinctly enhanced or decreased the expression of these microRNAs, i.e. suggesting promotion or inhibition of cardiomyogenesis. Furthermore, Fgf2 and Fgf8, but not Bmp2 or Bmp4, can induced expression of cardiomyocyte terminal differentiation marker in endocardial but not in epicardial cells.

**FIGURE 10:**
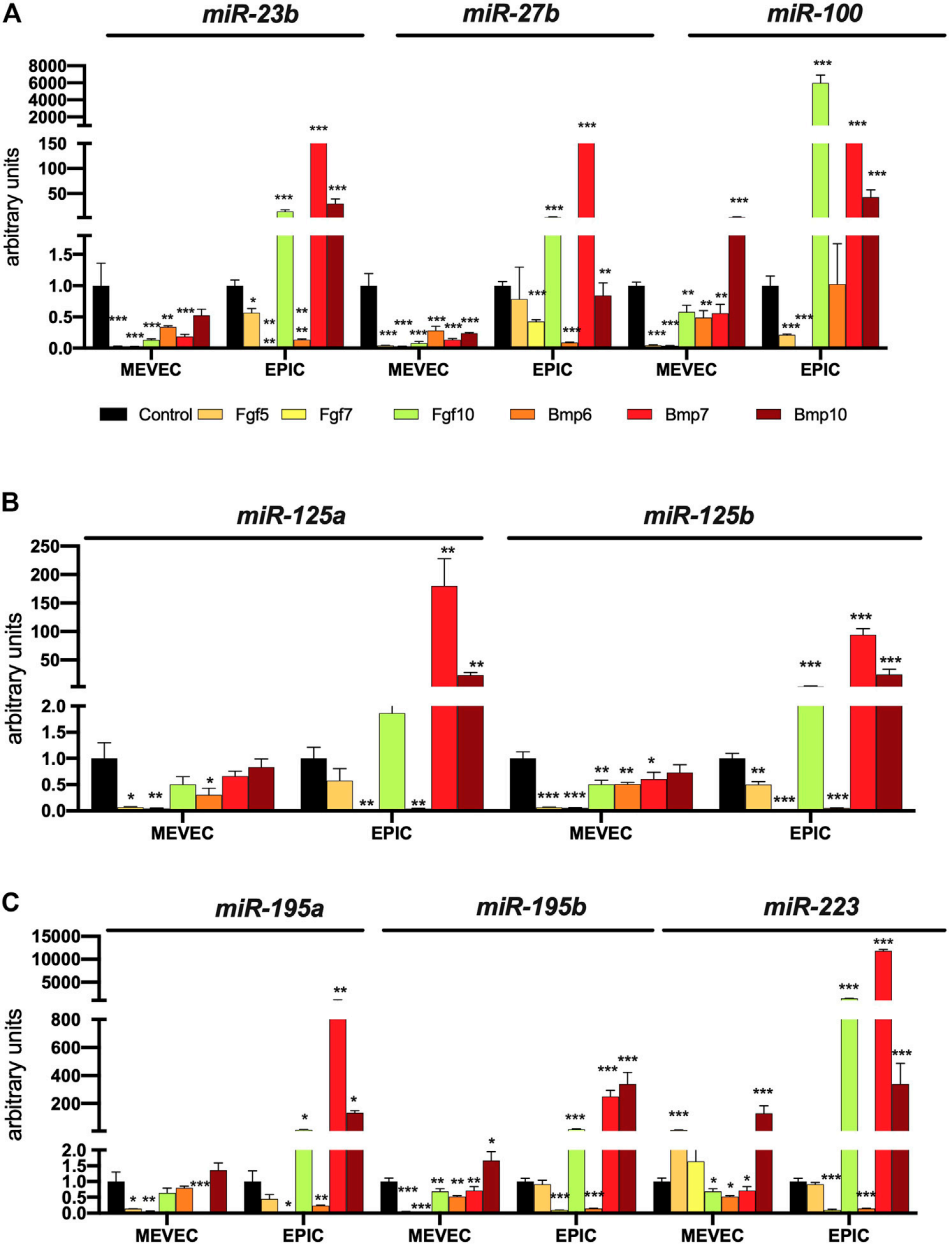
Panel **(A)** RT-qPCR analyses of miR-23b, miR-27b and miR-100 expression after control, Bmp6, Bmp7, Bmp10, Fgf5, Fgf7 and Fgf10 treatments in MEVEC (endocardial) and EPIC (epicardial) cell lines, respectively. Panel **(B)** RT-qPCR analyses of miR-125a, miR-125b and miR-146b expression after control, Bmp6, Bmp7, Bmp10, Fgf5, Fgf7 and Fgf10 treatments in MEVEC (endocardial) and EPIC (epicardial) cell lines, respectively. Panel **(C)** RT-qPCR analyses of miR-195a, miR-195b and miR-223 expression after control, Bmp6, Bmp7, Bmp10, Fgf5, Fgf7 and Fgf10 treatments in MEVEC (endocardial) and EPIC (epicardial) cell lines, respectively. *p < 0.05, **p < 0.01, ***p < 0.001, ****p < 0.0001.

Additionally we also tested whether novel Bmp and Fgf family members with enhanced expression in PE/EE transition, might similarly modulate the expression of these microRNAs and/or distinct lineage markers. All growth factor tested significantly decreased the expression of miR-23b, miR-27b and miR-100 in MEVEC cells, except Bmp10 that enhanced expression of miR-100. Curiously, expression of these microRNAs is enhanced in EPIC cells for Fgf10, Bmp7 and Bmp10, except for miR-27b that is decreased by Bmp10 administration **(** Figure 10A). Similarly, all growth factor tested decreased the expression of miR-125a and miR-125b in MEVEC cells or show no significant differences whereas Bmp7 and Bmp10 significantly up-regulated them in EPIC cells. In addition, Fgf10 also upregulates miR-125b in EPIC cells (Figure 10B). On the other hand, miR-195a and miR-195b are significantly downregulated by all growth factors tested in MEVEC except for Bmp10 does not alter miR-195a expression and increased miR-195b expression while miR-223 is significantly upregulated by Fgf5 and Bmp10 and down-regulated by Fgf10, Bmp6 and Bmp7 in MEVEC cells (Figure 10C). Importantly, Fgf10, Bmp7 and Bmp10 significantly upregulate miR-195a, miR-195b and miR-223 in EPIC cells, while the other growth factors tested decreased or did not modify their expression in this cell line (Figure 10C).

**FIGURE 11:**
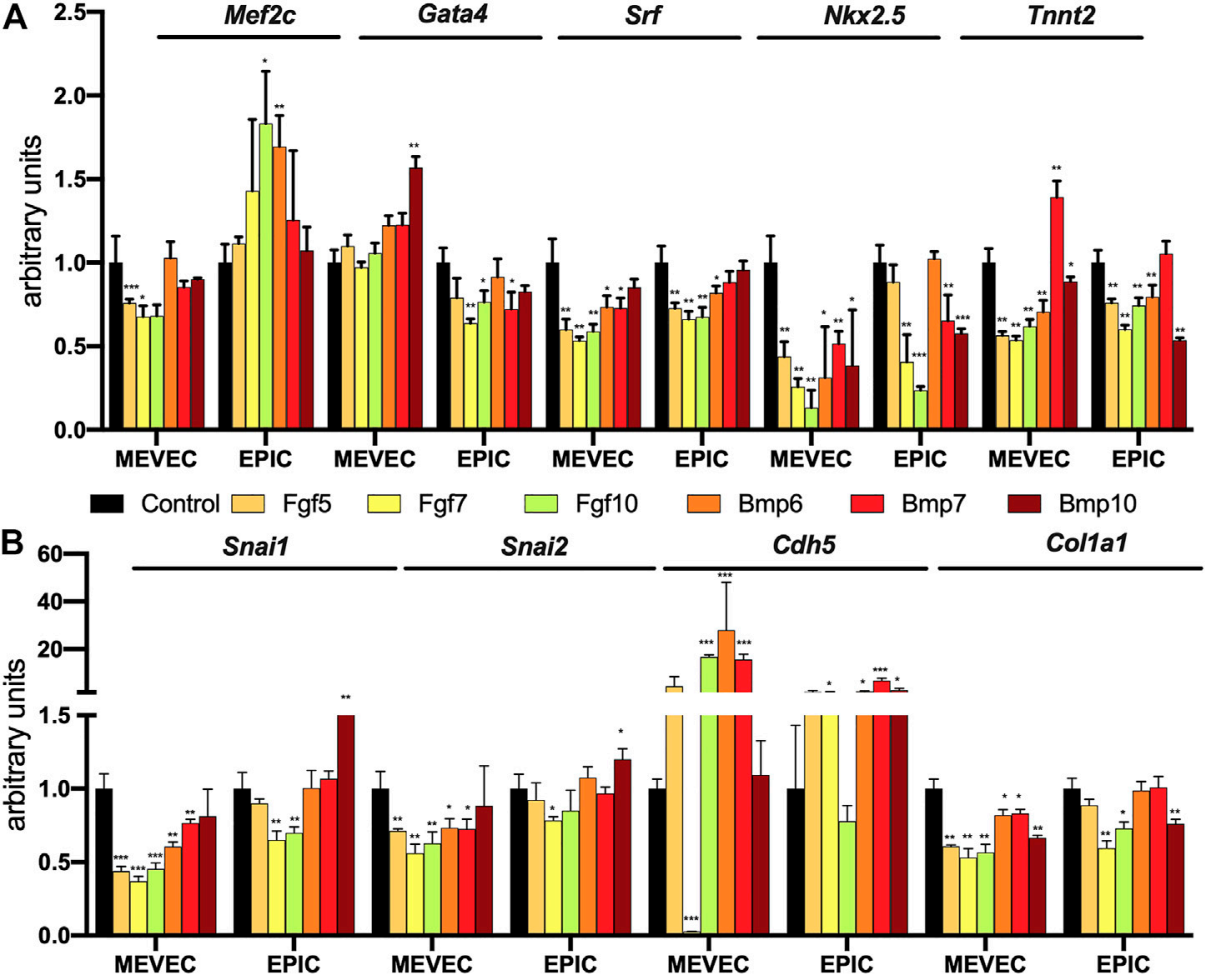
Panel **(A)** RT-qPCR analyses of Mef2c, Nkx2.5, Srf, Gata4 and Tnnt2 expression after control, Bmp6, Bmp7, Bmp10, Fgf5, Fgf7 and Fgf10 treatments in MEVEC (endocardial) and EPIC (epicardial) cell lines, respectively. Panel **(B)** RT-qPCR analyses of Snai1, Snai2, Cdh5 and Col1a1 expression after control, Bmp6, Bmp7, Bmp10, Fgf5, Fgf7 and Fgf10 treatments in MEVEC (endocardial) and EPIC (epicardial) cell lines, respectively. *p < 0.05, **p < 0.01, ***p < 0.001, ****p < 0.0001.

Lineage marker analyses after Bmp and Fgf administration showed that none of them is capable of enhancing early cardiomyogenic lineage markers in MEVEC cells, and most of them lead to significant downregulation, except for Bmp7 administration that resulted in significant upregulation of terminal cardiomyocyte differentiation marker Tnnt2 (Figure 11A). Within EPIC cells, only upregulation of early cardiomyocyte differentiation markers is observed, particularly Mef2c, but expression of Tnnt2 is either not altered or significantly downregulated by all growth factors tested.

Similarly, EMT instructive genes such as Snai1 and Snai2 are significantly down-regulated in MEVEC cells and EPIC cells, except for Bmp10 that enhanced Snai1 and Snai2 expression in EPIC cells. In line with this findings, Cdh5 is upregulated in all experimental conditions in both cell lines, particularly for Fgf10, Bmp6 and Bmp7 in MEVEC cells (Figure 11B) and Fgf7, Fgf10, Bmp6, Bmp7 and Bmp10 in EPIC cells. Fibrogenic marker analyses also demonstrate that all Bmp and Fgf treatments both cell types leads to downregulation of Col1a1 expression (Figure 11B). These data demonstrate that none of the Bmp and Fgf treatments reported herein lead to cardiomyocyte differentiation in epicardial cells and only Bmp7 is capable of inducing terminal differentiation in endocardial cells. In addition, EMT and fibrogenic differentiation are similarly halted in both cell lines by all growth factors analyzed.

## Discussion

The role distinct microRNAs during cardiovascular development has been widely demonstrated (Xin et al., 2013; Wojciechowska et al., 2017; Kalayinia et al., 2021). Conditional deletion of *Dicer* in the developing heart (Saxena and Tabin, 2010) or even specifically in the developing epicardium (Singh et al., 2011) leads to cardiovascular defects. Furthermore miR-1 mutants (Zhao Y. et al., 2007; Heidersbach et al., 2013) and miR-126 (Fish et al., 2008) are embryonic lethal due to cardiovascular alterations. Additional evidences on the role of distinct microRNAs during cardiogenesis have been reported for miR-130 in cardiomyogenic mesoderm determination (Lopez-Sanchez et al., 2015b), and for miR-23 and miR-199 in endocardial cushions formation (Lagendijk et al., 2011; Bonet et al., 2015). We have recently demonstrated that administration of miR-195 and/or miR-223 in enhances PE/ST-derived cardiomyogenesis in chicken (Dueñas et al., 2020). However, we demonstrated herein that such inductive roles are not conserved in mouse PE/ST explants. Such discrepancies might be related to the distinct morphogenetic events during PE development between mouse and chicken (Shulte et al., 2007) and thus that distinct signaling pathways that are involved (Shulte et al., 2007).

Bmp and Fgf have been reported to play essential roles in multiple aspects of embryogenesis (Ornitz and Itoh, 2015; Ornitz and Marie, 2015; Wu et al., 2016; Graf et al., 2016; Salazar et al., 2016; Zinski et al., 2018; Xie et al., 2020; Mossahebi-Mohammadi et al., 2020). In particular, during heart development, Bmp have been involved in early cardiogenic precursor determination (Lopez-Sanchez et al., 2002; López-Sánchez and García-Martínez, 2011) and also in later developmental states of myocardial growth and/or valvular development (Delot, 2003; Chen et al., 2004; Kruithof et al., 2012; Garside et al., 2013). Within the PE/ST development, distinct Bmp and Fgf have been reported during chicken development, demonstrating that Bmp2 and Bmp4 enhance cardiomyocyte commitment of precardiac mesoderm while Fgf2 and Fgf8 provide signaling cues to direct these cells into the PE lineage (Kruithof et al., 2006). We recently demonstrate that microRNAs involved in PE/ST-derived cardiomyogenesis are distinctly regulated by Bmp2/Bmp4, Fgf2/Fgf8 (Dueñas et al., 2020). Given the fact that miR-195 and miR-223 administration does not enhanced PE/ST cardiomyogenesis in mice, thus it might be plausible that such regulatory pathway is impaired in mice. Our data demonstrate primarily Fgf2 and Fgf8 enhanced expression of miR-195a, miR-195b and miR-223, while only Bmp2 enhanced miR-195b and Bmp4 enhanced miR195a, yet in any case early or terminal cardiomyocyte differentiation is increased in mouse PE/ST explants. Thus, these data suggest that alternative pathways might be involved regulating these PE/ST-derived cardiomyogenesis enhancing microRNAs. Curiously, neither Bmp2/Bmp4 and/or Fgf2/Fgf8 elicited modulation on EMT and fibrogenic markers, supporting a limited role of these growth factors directing key developmental processes during PE/ST development in mice.

Distinct regulatory roles were observed in epicardial and endocardial cell lines. Bmp2/Bmp4 and Fgf2/Fgf8 distinctly modulate the expression of microRNAs that inhibit PE/ST-derived cardiomyogenesis (miR-23b, miR-27b and miR-100), preferentially up-regulating them in MEVEC cells while down-regulating them in EPIC cells. For those microRNAs that mildly enhance early cardiomyogenesis markers (miR-125a, miR-125b and miR-146b) Bmp2 leads to upregulation in MEVEC but not in EPIC cells while Bmp4 enhanced it in both cell lines. Surprisingly, those microRNAs that enhance cardiomyocyte terminal differentiation (miR-195a, miR-195b and miR-223) were similarly up-regulated in both cell lines by Bmp2 and Bmp4 but up-regulated only in endocardial cells by Fgf2 and Fgf8. In line with these findings, terminal cardiomyocyte differentiation is elicited exclusively in endocardial cells by Fgf2 and Fgf8. In addition EMT induction is similarly increased in both cells lines by Bmp2 and Bmp4, in line with previous findings in other embryonic contexts (Ma et al., 2005; Inai et al., 2008; Cai et al., 2011; Townsend et al., 2011; Inai et al., 2013; Richter et al., 2014), while fibrogenesis is exclusively increased by Fgf2 anf Fgf8 in endocardial but not in epicardial cells.

As revealed by RNAseq analyses during PE development in mice, several additional Bmp and Fgf family members (Bmp6, Bmp7, Bmp10, Fgf5, Fgf7 and Fgf10). are differentially expressed, suggesting a plausible role during PE development in mice. Curiously, Fgf8 expression was not detectable in PE/EE mouse tissues, suggesting that it might not be relevant for mouse PE development, in line with our findings *in vitro*.

Bmp6 and Bmp7 are required for endocardial cushion formation during heart development (Kim et al., 2001), yet no evidences of their functional role and/or expression have been reported to date in the PE. The role of Bmp10 have been reported in different aspect of cardiac development (Neuhaus et al., 1999; Chen et al., 2004; Somi et al., 2004; Teichmann and Kessel, 2004; Huang et al., 2012; Capasso et al., 2020), yet no role in PE development is provided to date. While no evidences have been reported for Fgf5 and Fgf7 during heart development or PE development, Fgf10 have been implicated during both heart development (Kelly et al., 2001; Chan et al., 2010; Watanabe et al., 2010; Vega-Hernández et al., 2011; Rochais et al., 2014; Hubert et al., 2018) and PE formation (Torlopp et al., 2010). Thus, we identified herein novel Bmp and Fgf family members with potential implication in mouse PE/ST development.

Importantly, administration of different Fgf and Bmp growth factors to murine PE/ST explants distinctly modulate the expression of microRNAs that inhibit PE/ST-derived cardiomyogenesis (miR-23b, miR-27b and miR-100) as well as those that mildly enhance early cardiomyogenesis markers (miR-125a, miR-125b and miR-146b). However, none of them significantly up-regulate those microRNAs that enhance cardiomyocyte terminal differentiation (miR-195a, miR-195b and miR-223). In line with these results, no terminal differentiation up-regulation is observed in PE/ST explants. On the other hand, EMT induction is documented after Bmp6 and Bmp10 administration and severely blocked by Fgf5, Fgf7 and Fgf10. Overall, these data support the notion that these growth factors do not play a functional role enhancing cardiomyogenesis in the mouse PE and provide novel insights into the plausible role of Bmp10, Fgf5, Fgf7 and Fgf10 regulating cardiovascular EMT, as reported for, e.g. Fgf10, in other biological contexts (Abolhassani et al., 2014; Farajihaye Qazvini et al., 2019).

microRNA regulation by Fgf and Bmp family members is distinctly observed in epicardial and endocardial cell lines. Fgf10, Bmp7 and Bmp10 resulted in sustained upregulation of all cardiomyogenic inductive and inhibiting microRNAs in EPIC but not in MEVEC cells, supporting the notion of complex genetic post-transcriptional regulatory mechanisms driven by these growth factors. In this context, the end result is that neither early (with the exception of Mef2c) nor late cardiomyogenic terminal differentiation is achieved in any of the 2 cell types, except for Bmp7 in MEVEC cells. Thus, these data suggest that Bmp7 can be taken over the regulatory roles of other Bmp family members during PE/ST mouse development, yet it is highly intriguing that such effects are only observed in endocardial but not in epicardial cells. Additional experiments are required to fully understand these discrepancies.

Moreover, all Bmp and Fgf family members tested inhibited EMT, as Snai1 and Snai2 are preferentially downregulated and Cdh5 is upregulated, with the exception of Bmp10 in epicardial cells. Thus, these data further support the previous findings in PE/ST explants, highlight the plausible novel role of Fgf5, Fgf7, Fgf10, Bmp6, Bmp7 and Bmp10 in EMT regulation.

In summary, we demonstrated herein that PE/ST-derived cardiomyogenesis is distinctly regulated during chicken and mouse development. Distinct Bmp and Fgf family members play fundamental roles regulating miR-195/miR-223 expression in chicken PE/ST, that ultimately lead to enhanced cardiomyogenesis in chicken (Dueñas et al., 2020). However, such regulatory effects are not conserved in mouse PE/ST explants.

## Data Availability

RNAseq data were uploaded into Gene Expresssion Onmibus platform with accession number GSE189344. https://www.ncbi.nlm.nih.gov/geo/query/acc.cgi?acc=GSE189344
