# Supplementary material for: The Role of Bmp- and Fgf Signaling Modulating Mouse Proepicardium Cell Fate
Source: Front Cell Dev Biol. 2022 Jan 4;9:757781. doi: 10.3389/fcell.2021.757781 (PMC8763981; doi:10.3389/fcell.2021.757781)
Supplement: Supplementary file 1 [file DataSheet1.PDF]

Gene	Forward (5´-3´)	Reverse (5´-3´)
Mef2c	TCCACCTCGGCTCTGTAACT	CAGCTGCTCAAGCTGTCAAC
Nkx2.5	TTGGCGTCGGGGACTTGAAC	GGTGGGTGTGAAATCCGAGGGAC
Srf	GCTCAATGCCTTCTCTCAGG	CCCTATCACAGCCATCTGGT
Gata 4	TCTCACTATGGGCACAGCAG	CGAGCAGGAATTTGAAGAGG
Tnnt2	TTCGACCTGCAGGAAAAGTT	GCACAGCTTTGACGAGAACA
Snai1	CTTGTGTCTGCACGACCTGT	AGTGGGAGCAGGAGAATGG
Snai2	CATTGCCTTGTGTCTGCAAG	GATGTGCCCTCAGGTTTGAT
Cdh5	TGCATCCTCACCATCACAGT	AGTGACCAACTGCTCGTGAA
Col1a1	CACTGCAAGAACAGCGTAGC	GACTGTCTTGCCCCAAGTTC
Wt1	ACCATCTGAAGACCCACACC	TCTGATGCATGTTGTGATGG
Tcf21	CGCTCACTTAAGGCAGATCC	TCACCACTTCCTTCAGGTCA
Tbx18	GGCCATCATTACGGACTCTC	GGAGCAAGAGGATCCAGACA

Supplementary Table 1. Nucleotide sequences of the oligonucleotides used for qPCR analyses
